# Omega-6 polyunsaturated fatty acids and adiposity in the UK Biobank Cohort: a cross-sectional and longitudinal prospective analysis

**DOI:** 10.1017/S0007114526107430

**Published:** 2026-05-06

**Authors:** Heidi T. M. Lai, Jason Westra, Evan De Jong, Nathan L. Tintle, Martha A. Belury, William S. Harris

**Affiliations:** 1 Fatty Acid Research Institute, Sioux Falls, SD, USA; 2 Dordt University, Sioux Center, IA, USA; 3 Department of Human Sciences, Ohio State University, Columbus, OH, USA; 4 OmegaQuant Analytics, Sioux Falls, SD, USA; 5 Department of Internal Medicine, Sanford School of Medicine, University of South Dakota, Sioux Falls, SD, USA

**Keywords:** omega-6, Linoleic acid, Obesity, Waist circumference, Anthropometry, Cohort, longitudinal

## Abstract

The role of *n*-6 PUFA, especially linoleic acid (LA), in adiposity remains contested. While clinical interventions suggest improved body composition with higher LA intake, observational evidence using dietary data is inconsistent, and few studies consider circulating fatty acids or longitudinal changes in adiposity. Using multivariable linear models, we evaluated cross-sectional and longitudinal associations between *n*-6 PUFA and waist circumference (WC), weight and whole-body fat mass (FM) in the UK Biobank Cohort. Cross-sectionally (*n* 272 587, 54 % female, mean age 57 years), higher circulating LA was inversely associated with WC, weight and FM. Participants in the highest *v*. lowest quintile of LA had significantly smaller WC (–11·04 (–11·17, –10·91) cm), lower weight (–11·77 (–11·92, –11·62) kg) and lower FM (–7·87 (–7·97, –7·77) kg). Associations for total *n*-6 were generally consistent with those for LA. Conversely, non-LA *n*-6 was positively associated with WC (1·46 (1·32, 1·61) cm), weight (2·41 (2·25, 2·58) kg) and FM (1·81 (1·69, 1·92) kg). Longitudinal analyses (*n* 58 335, 51 % female, mean age 55 years) largely corroborate these patterns, with annual changes in WC, weight and FM inversely associated with LA and positively associated with non-LA *n*-6. Higher circulating LA, but not non-LA *n*-6, was associated with lower WC, weight and FM both cross-sectionally and longitudinally. Our findings potentially support dietary recommendations to promote LA-rich oils. Divergent associations between LA and non-LA *n*-6 caution against treating *n*-6 PUFA as a homogeneous group. Examining the distinct health effects of individual non-LA *n*-6 is warranted.

Obesity affects 890 million adults worldwide^(1)^ and is a major risk factor for cardiometabolic disease^(2)^, contributing to more than 1·6 million premature deaths each year globally^(3)^. By 2035, the global economic burden attributable to obesity is projected to exceed $4 trillion annually (USD)^(4)^. As suboptimal diet is a modifiable risk factor for obesity^(5)^, identifying determinants of adiposity is an urgent public health priority.

Linoleic acid (LA), an essential *n*-6 PUFA, accounts for 85–90 % of *n*-6 PUFA intake in the Western diet and is abundantly sourced from seed oils (e.g. soyabean, corn, cottonseed oils), nuts and seeds^(6,7)^. The majority of non-LA *n*-6 PUFA in the diet and the blood consist of arachidonic acid (AA)^(6)^. Others such as dihomo-gamma-linolenic acid, *γ*-linolenic acid, adrenic acid and Osbond acid are predominantly determined by metabolic processes, though AA is minimally available in poultry, eggs and meat sources^(7)^. Although LA is well studied for its cardiometabolic effects^(8)^, its role in body-weight regulation remains contested^(9)^.

Several arguments have been raised that point to potential adverse effects of all *n*-6 PUFA, or LA in particular. First, ecological observations show that rising *n*-6 PUFA levels in the diet (and a parallel decline in saturated fats) coincided with increased cardiometabolic disease rates in the early 20th century^(10)^. Second, LA serves as a precursor to AA, which is a substrate for several pro-inflammatory eicosanoids^(11)^, fuelling speculation that a high LA intake may promote chronic systemic inflammation and obesity via its conversion to AA^(7)^. More recently, all *n*-6 PUFA (including LA) attracted adverse publicity related to their association with industrial food processing^(12,13)^. In terms of mechanistic concerns, studies in animal models (C57BL/6j mice) demonstrated that elevated levels of dietary LA increased endocannabinoid production, promoted weight gain and impaired insulin signalling^(14,15)^. Specifically, a diet comprised of 22·5 % energy intake from LA induced greater weight gain than saturated fat diets, despite the absence of hypothalamic inflammation^(15)^. However, this dose far exceeds typical human intakes, and the applicability of these findings to humans remains questionable.

In contrast, clinical interventions generally report reduced systemic inflammation and improved body composition with the addition of LA to the diet^(9,16–19)^, especially in lean mass^(17,19)^. Observational cohorts also link higher levels of circulating total *n*-6 PUFA, LA and, in some cases, AA, to anti-inflammatory profiles^(20–22)^. In studies incorporating anthropometric measures, circulating LA was positively associated with lean tissue volume^(23)^ and skeletal muscle mass^(24)^, inversely associated with trunk adipose mass^(23)^ and abdominal obesity^(25)^, although associations for weight gain and BMI are mixed^(23,26,27)^. Findings are less consistent for the associations between AA and BMI^(26,28)^, while other non-LA *n*-6 PUFA have not been individually studied. LA was also inversely associated with metabolic syndrome^(29,30)^, suggesting that at population-relevant exposures, higher LA is unlikely to promote and might even attenuate weight gain.

Despite this body of work, some clarifications are warranted. Findings from studies that rely on dietary self-report methods are less consistent, reporting largely null associations between *n*-6 overall or LA in particular with anthropometric measurements^(28,31–34)^, potentially reflecting exposure misclassification. Furthermore, few studies have incorporated repeated measures to assess baseline levels and longitudinal changes over time. Large-scale studies using objective biomarkers of LA status, particularly in relation to adiposity outcomes, are also sparse. To address these limitations from prior studies, we examined the cross-sectional and longitudinal relationship between circulating LA levels in relation to body weight and adiposity outcomes in the UK Biobank (UKBB), a large, prospective cohort.

## Methods

### Study population

The UKBB is a prospective, population-based cohort of ∼500 000 individuals recruited between 2007 and 2010 at assessment centres across England, Wales and Scotland. Baseline data were collected using questionnaires, biological samples and physical measurements. Ongoing longitudinal monitoring occurs via a mix of in-person measurements, in-person and online questionnaires, as well as nearly real-time electronic medical record and death registry integration^(35,36)^. To be included, participants needed to have data on fatty acid and all covariates used in the analysis. Of 502 128 subjects in the UKBB, FA data were available on a random sample of 274 003. After removing 1416 individuals with incomplete information on covariates (anthropometric measures, *n* 1090; Townsend Deprivation Index^(37)^, *n* 326), the final sample available for cross-sectional analysis was 272 587 (online Supplementary Figure 1). For longitudinal associations, we used the subsample of 98 927 individuals who attended at least one follow-up and used the first instance of those who attended multiple follow-ups. Of these 98 927 individuals, a random sample of 59 394 had baseline fatty acids. After removing an additional 1059 individuals for missing covariate data, our final sample of 58 335 individuals included follow-up measures from the repeat assessment (*n* 17 906), the imaging visit (*n* 40 192) or the first repeat imaging visit (*n* 237)^(38)^ (online Supplementary Figure 1).

### Exposure

Our two primary exposures are plasma LA and non-LA *n*-6 (each expressed as a percentage of total plasma FA). The secondary exposure is total *n*-6. The level of total *n*-6 and LA was determined on baseline plasma samples using NMR (Nightingale Health)^(39)^; analytical performance characteristics have been reported^(40)^. Non-LA *n*-6 is computed as the difference between total *n*-6 and LA.

### Outcomes

Our primary outcomes are waist circumference, weight (both of which are measured rather than estimated) and whole-body fat mass (as it is the dominant compartment among other estimated outcomes). Secondary outcomes included BMI, whole-body fat-free mass, trunk fat mass and trunk fat percent. For cross-sectional associations, we relied on anthropometric measurements taken at baseline (2006–2010). For analyses that explored changes over time, we relied on follow-up measurements taken in 2012–2013 during the first repeat assessment, 2014 and onwards for an imaging visit and 2019 and onwards for the first repeat imaging visit. Waist circumference was taken by a Seca 200 cm tape measure, standing height by Seca 240 cm height measure, and the remaining measurements were collected by a Tanita BC418MA body composition analyser (https://tanita.eu/understanding-your-measurements), all via standard protocol (https://biobank.ctsu.ox.ac.UK/ukb/ukb/docs/Anthropometry.pdf).

### Covariates

Pre-planned covariates were considered based on biological interest, current or previously observed associations with *n*-6 or adiposity outcomes and meaningful changes in the exposure risk estimate (±5 %). Information on all reported sociodemographic characteristics, diet and lifestyle factors was collected via a touchscreen questionnaire. The electronic questionnaire and other resources can be found on the UKBB website (https://www.ukbiobank.ac.UK/resources/). In brief, pre-planned covariates included in our models were age, sex, education, Townsend Deprivation Index, ethnicity, physical activity and levels of DHA. The inclusion of DHA in the model allows us to assess the association between *n*-6 PUFA and adiposity independent of the influence of DHA, as *n*-3 PUFA share metabolic pathways with *n*-6 PUFA. Details of covariates used and their corresponding ID in the UKBB database can be found in online Supplementary Table 1.

### Statistical analysis

Sample characteristics were summarised using standard approaches (mean/sd; *n*/%). The associations between the exposures of interest (LA and non-LA *n*-6 PUFA) and cross-sectional outcome measures were assessed with multivariable linear models, which adjusted for relevant covariates. Longitudinal linear models, with findings reported as standardised betas (95 % CI), predicted changes in outcome measures divided by the length of time (years) between measurements to account for person-to-person differences in exposure time and adjusting for relevant covariates. Associations are expressed as a change in the outcome measure per interquintile range (IQ_5_R, defined as 90th minus 10th percentiles of each exposure of interest) or per quintile (Q; relative to the lowest quintile (Q1)) and via a linear trend across fatty acid quintiles. For longitudinal models, additional adjustments for baseline weight, baseline waist circumference and baseline whole-body fat mass were included. Exploratory analyses used restricted cubic splines to test for potential non-linearity *v*. linear models between LA and the three primary cross-sectional and three primary longitudinal outcomes. Interaction terms between continuous LA (IQ_5_R) and sex or (separately) age decade (40–50, 50–60, 60–70) were added to each model (cross-sectional and longitudinal) to test for potential sex or age modification of the LA–outcome relationships. Additionally, for the relationship between LA and whole-body fat mass and whole-body fat-free mass, mutual adjustments were included as a sensitivity analysis to control for potential confounding. A significance level of 0·05 was used for all analyses.

## Results

### Participant characteristics

We evaluated a total of 272 587 participants for the cross-sectional analysis and 58 335 participants for the longitudinal analysis (Table 1). Distributions of sex, ethnicity, education, Townsend Deprivation Indices and physical activity were similar between the cross-sectional and longitudinal sub-cohorts. About half were female, nearly all were White and just about half reported a college education. Participants also tended to be slightly overweight at baseline, with a general reduction in weight and whole-body fat-free mass during follow-up. Correlations between anthropometric measures are reported in online Supplementary Table 2.

**Table 1.** Participant characteristics of the UK Biobank Table 1 long description.

	Cross-sectional		Longitudinal	
Variable	Frequency	%	Frequency	%
Sex				
Female	147 176	54	30 198	52
Male	125 411	46	28 137	48
Age (years)				
Mean	56·6		55·4	
sd	8·08		7·63	
Ethnicity				
White	258 175	94·7	56 425	96·7
Asian	5627	2·1	765	1·3
Black	3807	1·4	374	0·6
Missing	1205	0·4	164	0·3
Other	3773	1·4	607	1·0
Education				
College	145 529	53·4	37 403	64·1
High school	76 191	28·0	16 265	27·9
Less than high school	47 735	17·5	4434	7·6
Missing	3132	1·1	233	0·4
Townsend score				
Mean	–1·36		–1·93	
sd	3·07		2·73	
Physical activity				
Lowest	60 661	22·3	13 154	22·5
Low	62 012	22·7	14 673	22·2
High	62 299	22·9	15 071	25·8
Highest	63 192	23·2	12 924	22·2
Missing	24 423	9·0	2513	4·3
	**Mean**	**sd**	**Mean**	**sd**
*Fatty acid measurements* *(% total fatty acid)*				
Linoleic acid (LA)	28·9	3·44	29·3	3·33
*n*-6 PUFA	37·9	3·63	38·2	3·50
*n*-6: non-LA	8.96	1·91	8·92	1·86
DHA	2·00	0·68	2·05	0·67
*n*-3 PUFA	4·38	1·55	4·46	1·55
*n*-3: non-DHA	2·39	1·01	2·40	1·01
*Anthropometric measures (baseline)*				
Standing height (cm)	168·5	9·26	169·6	9·15
Weight (kg)	78·1	15·9	77·2	15·1
BMI (kg/m^2^)	27·5	4·78	26·8	4·36
Waist circumference (cm)	90·3	13·5	88·4	12·8
Whole-body fat-free mass (kg)	53·3	11·5	53·7	11·4
Whole-body fat mass (kg)	24·9	9·53	23·5	8·80
Trunk fat mass (kg)	13·8	5·16	13·1	4·86
Trunk percent fat (%)	31·2	8·00	30·0	7·80
Anthropometric measures (change per year)^*^				
Weight (kg)			–0·08	0·72
BMI (kg/m^2^)			0·00	0·25
Waist circumference (cm)			0·14	0·94
Whole-body fat-free mass (kg)			–0·16	0·36
Whole-body fat mass (kg)			0·08	0·64
Trunk fat mass (kg)			0·05	0·39
Trunk percent fat (%)			0·11	0·65

* Paired *t* tests were conducted between baseline and repeated measures: all were statistically significantly different (*P* < 0·001), except for BMI (*P* = 0·135).

### Cross-sectional relationship between levels of n-6 and primary measures

**Figure 1.**
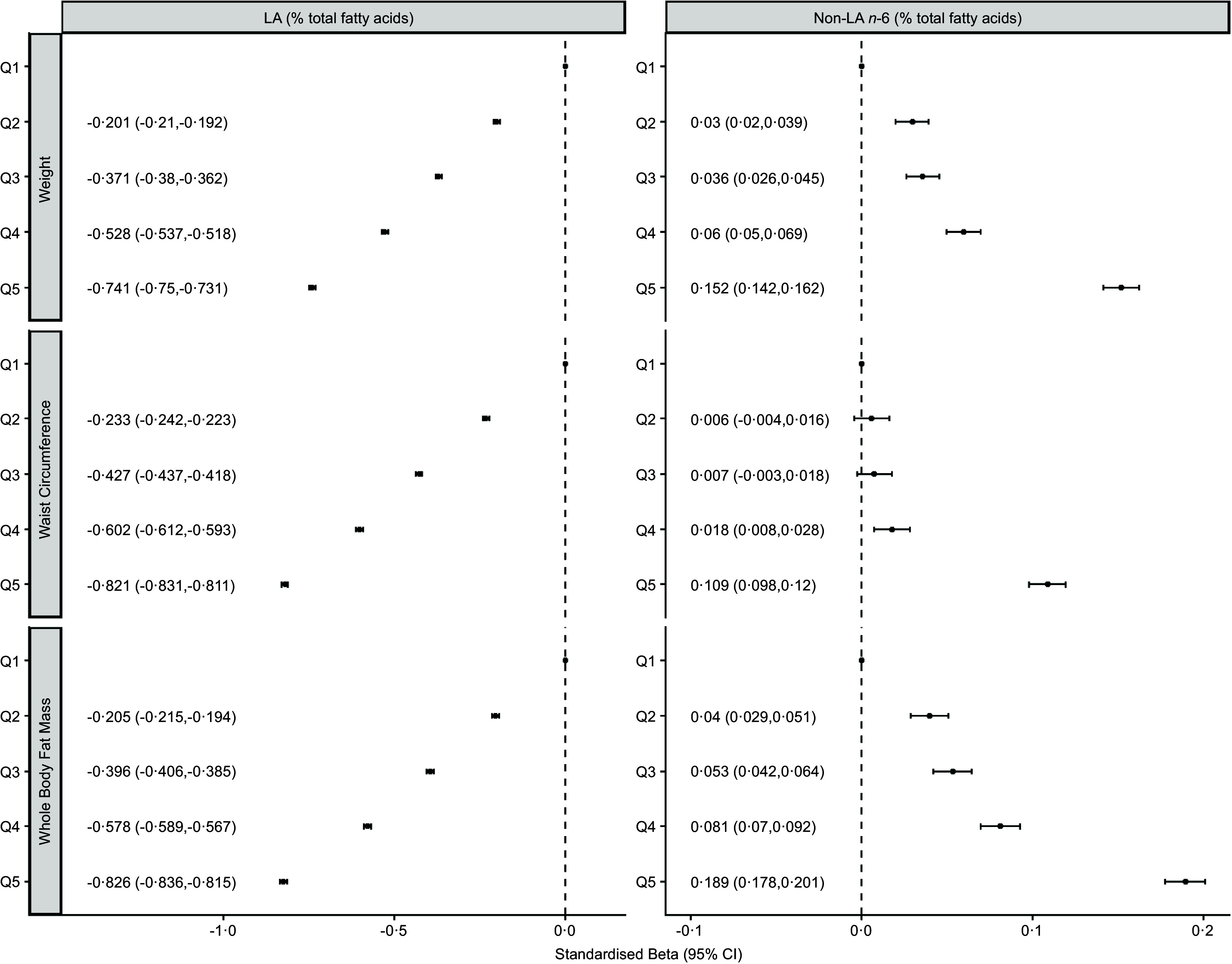
Fig. 1 long description. The cross-sectional association between plasma linoleic acid (LA, % total fatty acids), plasma non-linoleic acid *n*-6 PUFA (non-LA *n*-6) and weight (kg), waist circumference (cm) and whole-body fat mass (kg) in the UK Biobank. Models are adjusted for age, sex, ethnicity, standing height, education, Townsend scores, physical activity and levels of DHA.

In multivariable models, LA was inversely associated with waist circumference, weight and whole-body fat mass (Figure 1). In comparison with the lowest quintile, participants in the highest quintile had a statistically significant smaller waist circumference (–11·04 (–11·17, –10·91) cm), weighed less (–11·77 (–11·92, –11·62) kg) and had less whole-body fat mass (–7·87 (–7·97, –7·77) kg) (online Supplementary Table 3). While findings for total *n*-6 echoed those of LA (all *P* < 0·001) (online Supplementary Table 3), non-LA exhibited statistically significant associations in the opposite direction (Figure 1), though the magnitude of the relationships was smaller. Participants in the highest quintile had a slightly higher waist circumference (1·46 (1·32, 1·61) cm), were heavier (2·41 (2·25, 2·58) kg) and had more whole-body fat mass (1·81 (1·69, 1·92) kg) compared with the lowest quintile (online Supplementary Table 3). All associations remained robust and statistically significant when fatty acid levels were assessed continuously per IQ_5_R (online Supplementary Table 3).

### Changes in primary anthropometric measures over time

Similar to the results from our cross-sectional analysis, anthropometric measures decreased over time with higher levels of LA (Table 2). Waist circumference, weight and whole-body fat mass for participants in the highest quintile were lower by –0·11 (–0·13, –0·09) cm, –0·05 (–0·07, –0·03) kg and –0·04 (–0·06, –0·02) kg per year, respectively. Similarly, rates of change per year by IQ_5_R followed the results of analyses by quintiles, showing a statistically significant decrease in all three outcomes. Results for total *n*-6 followed those of LA (online Supplementary Table 4). For non-LA *n*-6, waist circumference, weight and whole-body fat mass were higher over time. Extreme-quintile differences are an increase of 0·09 (0·07, 0·12) cm, 0·13 (0·11, 0·14) kg and 0·12 (0·10, 0·14) kg per year, respectively, in line with findings per IQ_5_R (Table 2).

**Table 2.** The longitudinal association between linoleic acid and change in weight, waist circumference and whole-body fat mass in the UK Biobank Table 2 long description.

	Quintiles of fatty acid												
*Linoleic acid*	I	II		III		IV		V					
		*β*	95 % CI	*β*	95 % CI	*β*	95 % CI	*β*	95 % CI	*P*-trend^*^	Per IQ_5_R^†^		*P*
Total *n*	11 654	11 657		11 689		11 679		11 656			58 335		
Median (% total fatty acid)	25·0	27·8		29·5		31·1		33·3			29·5		
Waist circumference (cm)^‡^	0 (Ref)	−0·01	−0·04, 0·01	−0·04	−0·07, −0·02	−0·07	−0·10, −0·05	−0·11	−0·13, −0·09	< 0·001	−0·10	−0·12, −0·08	< 0·001
Total *n*	11 654	11 657		11 689		11 679		11 656			58 335		
Median (% total fatty acid)	25·0	27·8		29·5		31·1		33·3			29·5		
Weight (kg)^‡^	0 (Ref)	0·02	0·00, 0·04	0·01	−0·01, 0·03	0·00	−0·02, 0·01	−0·05	−0·07, −0·03	< 0·001	−0·03	−0·05, −0·02	< 0·001
Total *n*	8875	8805		8615		8504		8306			43 105		
Median (% total fatty acid)	25·0	27·7		29·5		31·1		33·3			29·4		
Whole-body fat mass (kg)^‡^	0 (Ref)	0·01	0·00, 0·03	0·00	−0·02, 0·02	−0·01	−0·03, 0·01	−0·04	−0·06, −0·02	< 0·001	−0·03	−0·05, −0·02	< 0·001
***Non-LA n-6***													
Total *n*	11 668	11 692		11 667		11 665		11 643			58 335		
Median (% total fatty acid)	6·6	7·9		8·8		9·8		11·3			8·8		
Waist circumference (cm)^‡^	0 (Ref)	0·02	0·00, 0·04	0·03	0·01, 0·06	0·03	0·01, 0·05	0·09	0·07, 0·12	< 0·001	0·09	0·07, 0·11	< 0·001
Total *n*	11 668	11 692		11 667		11 665		11 643			58 335		
Median (% total fatty acid)	6·6	7·9		8·8		9·8		11·3			8·8		
Weight (kg)^‡^	0 (Ref)	0·03	0·01, 0·05	0·05	0·04, 0·07	0·06	0·05, 0·08	0·13	0·11, 0·14	< 0·001	0·12	0·10, 0·13	< 0·001
Total *n*	8543	8602		8536		8613		8811			43 105		
Median (% total fatty acid)	6·7	7·9		8·8		9·8		11·3			8·8		
Whole-body fat mass (kg)^‡^	0 (Ref)	0·03	0·01, 0·05	0·06	0·04, 0·08	0·07	0·05, 0·09	0·12	0·10, 0·14	< 0·001	0·11	0·10, 0·13	< 0·001

LA, linoleic acid; IQ_5_R, interquintile range.

Models are adjusted for age, sex, ethnicity, standing height, baseline weight, baseline waist circumference, baseline whole-body fat mass, education, Townsend scores, physical activity and levels of DHA.

* *P*-trend is generated by assigning participants the median value in each quintile and then assessing quintiles as continuous variables.

† Interquintile range (IQ_5_R) is the difference between the first and fifth quintiles.

‡ Findings here are reported as non-standardised betas (95 % CI), the difference in comparison with the lowest quintile in terms of change per year. Negative values mean that the outcome of interest is smaller compared with the reference group (set as zero), while positive values indicate the opposite.

### Associations between n-6 levels and secondary outcomes

Cross-sectional associations with secondary outcomes (BMI, whole-body fat-free mass, trunk fat mass and trunk fat percent) are reported in online Supplementary Table 5. In brief, the direction of associations aligned with the primary outcomes; that is, a higher level of LA and total *n*-6 was linked to a lower BMI, whole-body fat-free mass, trunk fat mass and trunk fat percent, while non-LA *n*-6 demonstrated a statistically significant relationship in the opposite direction. For longitudinal associations, minor differences were present in comparison with primary outcomes (online Supplementary Table 6). For example, the association between LA and whole-body fat-free mass was neutral, and total *n*-6 was linked to increased levels of whole-body fat-free mass, but not trunk mass or trunk percent fat.

### Evidence of non-linearity

Restricted cubic splines showed evidence of non-linearity for the cross-sectional relationships between LA and all three primary outcomes (all three *P* < 0·001). At lower LA values (20–25 % of total fatty acids), associations showed a weaker inverse association with waist circumference, weight and whole-body fat mass (online Supplementary Figures 2–4), which strengthened as LA levels increased beyond 25 %, though it weakened again beyond 35 %. Similarly, all longitudinal models showed evidence of significant non-linearity (all three *P* < 0·05). Specifically, LA had the strongest relationships with yearly changes in waist circumference, weight and whole-body fat mass (online Supplementary Figures 5–7) at levels of LA beyond 32 %, with less evidence of a relationship for levels of LA less than 29 %.

### Interactions with age and sex

Cross-sectionally, the strength of the inverse relationship between LA waist circumference, weight and whole-body fat mass was statistically significantly stronger (all five *P* < 0·001) among participants who were younger or female (online Supplementary Table 7), in comparison with older or male participants, respectively. In longitudinal analyses, only weight change (with age; *P* < 0·05) and waist circumference change (with sex; *P* < 0·001) had statistically significant interactions. The stronger inverse relationship between LA and waist circumference was only present in participants who were female. Similarly, the stronger inverse association between LA and body weight was only present in those who were younger (online Supplementary Table 8).

### Sensitivity analysis

When whole‑body fat mass and fat‑free mass were mutually adjusted in the cross‑sectional models, associations were attenuated but remained statistically significant. Plasma LA was linked to a 3·43 kg lower whole‑body fat mass (95 % CI –3·49, –3·36) compared with a 6·93 kg lower fat mass (95 % CI –7·02, –6·85) without mutual adjustment. For fat‑free mass, the association shifted to –0·78 (–0·82, –0·74) kg *v*. –3·38 (–3·43, –3·33) kg without mutual adjustment. In longitudinal analyses, no appreciable differences were observed; for example, the estimated change in whole‑body fat mass was –0·03 (–0·05, –0·02) kg compared with –0·04 (–0·06, –0·03) kg without adjustment.

## Discussion

In our prospective cohort of over a quarter million participants, cross-sectional analyses showed that plasma LA and total *n*-6 PUFA were inversely associated with waist circumference, weight and whole-body fat mass. By contrast, higher levels of non-LA *n*-6 were linked to greater waist circumference, weight and whole-body fat mass, though the absolute values were small. Longitudinal analyses assessing annual change largely corroborate the cross-sectional findings. Findings for secondary outcomes (BMI, whole-body fat-free mass, trunk fat mass and trunk fat percent) are also aligned with primary outcomes. We also observed evidence for non-linearity in the associations between LA and waist circumference, weight and whole-body fat mass. All associations remain inverse and statistically significant after accounting for interactions with age and sex, although the strength of associations varied across subgroups.

Our findings show that circulating levels of plasma LA are inversely associated with waist circumference, weight and whole-body fat mass, supporting its potential protective role in body-weight regulation. Randomised controlled trials reinforce this biologic plausibility, demonstrating that LA may favourably influence adiposity through mechanisms such as improved insulin resistance^(16,23)^, maintenance of lean tissue^(16,23)^, reducing visceral fat^(17,41)^ and attenuating chronic inflammation^(23,42,43)^. Dietary fortification of LA directly increases the level of LA in the blood and tissue^(44)^, and diet is the primary source of LA, despite minor influences from genetics^(45)^ or biological interplay with *n*-3 PUFA^(46)^. Circulating LA is strongly associated with dietary LA, with the strongest dose–response association peaking at ≈8 % of total daily energy from dietary LA, though the association plateaus beyond 8 %^(47)^. Mechanistically, LA and its oxylipin metabolites act on G protein–coupled receptors and PPAR (PPAR*α*, PPAR*β*/*δ*, PPAR*γ*), modulating downstream pathways governing energy production and utilisation^(9)^. In addition, through the cytochrome P450 pathway, LA-derived vicinal diols (9,10-dihydroxy-9Z-octadecenoic acid, 12,13-dihydroxy-9Z-octadecenoic acid) are inversely associated with adiposity^(48,49)^, while levels of LA-derived epoxides (9(10)-epoxyoctadecenoic acid, 12(13)-epoxyoctadecenoic acid) are lower in subjects with metabolic syndrome^(50)^.

In contrast, plasma non-LA *n*-6 PUFA were positively associated with waist circumference, weight and whole-body fat mass, albeit with small effect sizes. Interpretation is challenging, as the non-LA *n*-6 fraction consists of several different *n*-6 PUFA, including the predominant AA, as well as dihomo-gamma-linolenic acid, *γ*-linolenic acid, adrenic acid and Osbond acid. While AA is known to exhibit pro-inflammatory effects, some of its metabolites (PG E2) are also inflammation resolvers (inhibition of TNF-*α*; inducing production of lipoxin A4)^(7)^. Similarly, oxylipins derived from other non-LA *n*-6, for example, dihomo-gamma-linolenic acid, may exert effects distinct from those of AA^(51)^. Hence, the findings on non-LA *n*-6 provide limited insight because the individual fatty acid associations cannot be evaluated.

Nevertheless, several takeaways can be drawn. If we consider findings for total *n*-6, the overall association is inverse, suggesting that the strong inverse association with plasma LA offsets the weak positive association of non-LA *n*-6 PUFA, resulting in an overall benefit. Conversely, it can also be said that investigating total *n*-6 masks the positive association between non-LA *n*-6 PUFA and adiposity. The divergence between LA and non-LA *n*-6 fractions challenges the common practice of pooling all *n*-6 PUFA into a single, global *n*-6 biomarker. Not all *n*-6 FA exert similar metabolic effects^(46)^. Hence, investigating individual *n*-6 FA, rather than treating *n*-6 PUFA as a homogeneous group, should be prioritised. Future investigations to elucidate how non-LA *n*-6 PUFA may influence adiposity, including their roles in eicosanoid production and inflammatory signalling, are warranted. Regardless, our findings potentially support current dietary guidelines^(52)^ that emphasise LA-rich foods, such as nuts, seeds, soyabeans, corn and sunflower oils, as part of a balanced diet for weight maintenance.

The non-linear associations between plasma LA and adiposity are interesting: cross-sectional analyses suggest threshold effects, with the strongest inverse associations observed up to ∼35 % of total fatty acids, beyond which additional LA confers little incremental benefit. This pattern is consistent with the behaviours of other FA^(53)^, where endogenous regulation limits saturation and excess accumulation^(7)^. In contrast, longitudinal analysis reveals a non-linear curve, suggesting that higher LA levels beyond this threshold may still confer benefits for changes in weight and whole-body fat mass, though replication in other studies is needed to confirm this finding. Fat mass and fat-free mass are physiologically interdependent, hence attenuation after mutual adjustment suggests that cross-sectional associations with each compartment may partly reflect shared variance rather than distinct biological pathways. This interdependence underscores the need for caution when interpreting fat mass-specific cross-sectional effects. The longitudinal findings (largely unchanged with or without mutual adjustment) likely provide a more reliable indication of the relationship between LA and change in adiposity. Interactions by age and sex indicate heterogeneity in effect sizes. In particular, stronger inverse associations in females could be linked to an upregulation in PUFA metabolism through female-related sex hormones^(54)^, underscoring the need to consider demographic stratification in future investigations.

Several studies have examined the association between *n*-6 PUFA and body anthropometrics, yet findings remain inconsistent. In analyses based on dietary intake, total *n*-6 PUFA, LA and AA were generally not associated with indices of adiposity, weight gain, body fat and relative fat mass^(28,31–34)^. An exception is a cross-sectional US national survey, which reported an inverse association between total *n*-6 intake and body fat percentage^(55)^. Studies using circulating biomarkers provide a somewhat clearer picture. One study that examined levels of cholesterol ester LA found significant inverse associations with sagittal abdominal diameter, waist circumference and waist:hip ratio^(25)^, supporting our main findings. By contrast, higher levels of circulating LA were positively linked to lean tissue volume^(18,23)^ and skeletal muscle mass^(24)^, which diverges from our secondary findings on fat-free mass. Though, notably, we estimated fat-free mass via bioimpedance rather than dual-energy X-ray absorptiometry. Only one study evaluated both cross-sectional and longitudinal associations^(26)^. LA, but not total *n*-6 PUFA or AA, was inversely associated with BMI cross-sectionally, whereas longitudinally (with a linear assumption), total *n*-6 PUFA were positively associated with BMI change, with null findings for LA and AA^(26)^. Such inconsistencies across studies likely reflect variation in exposure assessment (dietary *v*. circulating biomarkers), outcome measurement (dual-energy X-ray absorptiometry *v*. bioimpedance) and population characteristics. Against this backdrop, our study – the largest to date to our knowledge – extends and supports existing evidence by examining both cross-sectional and longitudinal associations of *n*-6 PUFA, particularly plasma LA, with seven anthropometric outcomes.

Several strengths warrant emphasis. First, our investigation benefited from a substantial sample size (over a quarter million cross-sectionally), which provided robust statistical power to detect associations. Second, the use of objective fatty acid biomarkers rather than self-reported intake minimised reporting bias, strengthening the validity of exposure assessment. Third, comprehensively characterised exposures, outcomes and potential confounding variables measured via standardised protocols enhanced comparability and reproducibility. Lastly, the availability of repeated anthropometric measurements allowed us to assess associations in both cross-sectional and longitudinal frameworks, providing insights into both baseline relationships and prospective changes over time.

Nonetheless, some limitations should be acknowledged. Non-LA *n*-6 FA were not individually quantified, precluding a more detailed investigation of their specific roles. We also did not explore the relationship between dietary intake of PUFA and circulating biomarkers. Bioimpedance is less precise compared with dual-energy X-ray absorptiometry, but these methods have been shown to be strongly correlated at a population level^(56)^. Participants were predominantly middle-aged to older adults, possibly limiting generalisability to younger populations. The UKBB also predominantly consists of White individuals and those with a higher socio-economic status; while these factors were accounted for in the analyses, some degree of selection bias may remain. As with all observational research, causality cannot be inferred, and residual confounding cannot be entirely excluded. However, our findings may provide insights to inform future randomised controlled trials and intervention studies.

In conclusion, higher levels of total *n*-6 PUFA, particularly plasma LA, were associated with smaller waist circumference and lower weight and whole-body fat mass in both cross-sectional and longitudinal analyses. In contrast, non-LA *n*-6 demonstrated associations in the opposite direction with weak effect sizes. Our findings potentially support dietary recommendations to include LA-rich oils in the diet. Furthermore, the divergent patterns between LA and non-LA *n*-6 highlight the limitations of relying on a global total *n*-6 biomarker, underscoring the need for future studies to investigate the specific determinants and potential health implications of individual non-LA *n*-6 fatty acids.

## Supporting information

10.1017/S0007114526107430.sm001 Lai et al. supplementary material Lai et al. supplementary material

## Supplementary material

For supplementary material/s referred to in this article, please visit https://doi.org/10.1017/S0007114526107430.

## Table 1. Long description

The table presents participant characteristics of the UK Biobank, comparing cross-sectional and longitudinal analyses. It includes variables such as sex, age, ethnicity, education, Townsend Deprivation Indices, physical activity, fatty acid measurements, and anthropometric measures. The table has 51 rows and 5 columns, with headers for Frequency, Percentage, and Mean with Standard Deviation. Key trends include similar distributions of sex, ethnicity, education, and physical activity between the two sub-cohorts. About half of the participants are female, nearly all are White, and just about half reported a college education. Participants tend to be slightly overweight at baseline, with a general reduction in weight and whole-body fat-free mass during follow-up. Notable data points include mean ages of 56.6 years for cross-sectional and 55.4 years for longitudinal participants, and mean body mass indices of 27.5 and 26.8 kilograms per square meter, respectively.

Navigate back to Table 1..

## Figure 1. Long description

The forest plot presents the cross-sectional association between plasma linoleic acid (LA, percentage of total fatty acids) and plasma non-linoleic acid n-6 PUFA (non-LA n-6) with weight in kilograms, waist circumference in centimeters, and whole-body fat mass in kilograms in the UK Biobank. The plot consists of two sets of vertical forest plots. The left set shows the association with LA, while the right set shows the association with non-LA n-6. Findings for each adiposity outcome, including weight, waist circumference, and whole-body fat mass, is divided into five quintiles (Q1 to Q5). The x-axis represents standardized beta values with 95 percent confidence intervals, ranging from negative one to positive zero for LA and from negative zero point one to positive zero point two for non-LA n-6. The y-axis lists the quintiles for weight, waist circumference, and whole-body fat mass. The models are adjusted for various factors including age, sex, ethnicity, standing height, education, Townsend scores, physical activity, and levels of DHA. The plot reveals an inverse association between LA and the measured variables, with higher quintiles showing more negative beta values. Conversely, non-LA n-6 shows a positive association, with higher quintiles displaying more positive beta values.

Navigate back to Figure 1..

## Table 2. Long description

The table presents data on the longitudinal association between linoleic acid and changes in weight, waist circumference, and whole-body fat mass in the UK Biobank. It is divided into quintiles of fatty acid, with rows for linoleic acid, non-LA n-6, and various anthropometric measures. Each quintile includes data on total participants, median percentage of total fatty acid, waist circumference, weight, and whole-body fat mass. The table shows that higher levels of linoleic acid are associated with decreases in waist circumference, weight, and whole-body fat mass over time. For participants in the highest quintile, waist circumference decreases by 0.11 centimeters per year, weight decreases by 0.05 kilograms per year, and whole-body fat mass decreases by 0.04 kilograms per year. Similar trends are observed for non-LA n-6, where waist circumference, weight, and whole-body fat mass increase over time. The extreme-quintile differences show an increase of 0.09 centimeters, 0.13 kilograms, and 0.12 kilograms per year, respectively. The table also includes P-values for trend and per IQ_5_R, indicating statistically significant decreases for LA and increases for non-LA in all three outcomes.

Navigate back to Table 2..
